# Supplementary material for: Kinetics and Fluid-Specific Behavior of Metal Ions After Hip Replacement
Source: Bioengineering (Basel). 2025 Dec 30;13(1):44. doi: 10.3390/bioengineering13010044 (PMC12838072; doi:10.3390/bioengineering13010044)
Supplement: Supplementary file 1 [file bioengineering-13-00044-s001.zip › bioengineering-3971050-supplementary.pdf]

Supplementary Materials

Scatter of Reported Data for Cobalt and Chromium

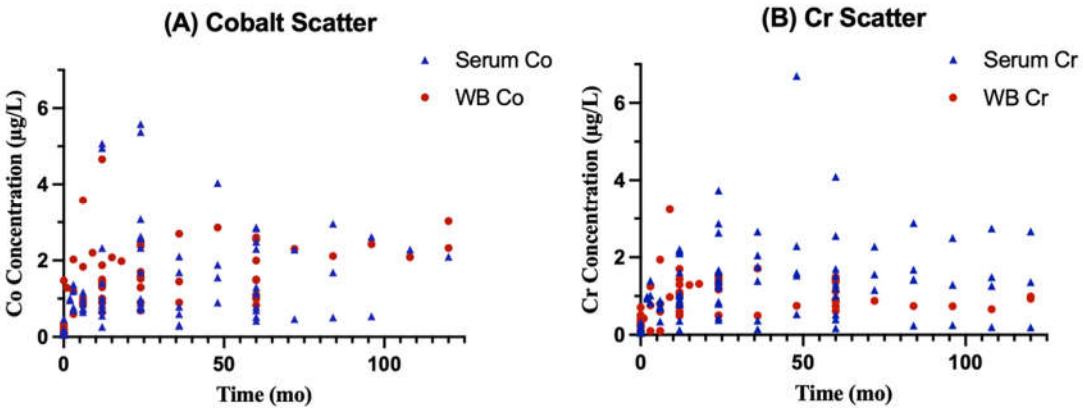

Figure S1. Reported concentrations (**A**) cobalt and (**B**) chromium measured in serum (blue triangles) and whole blood (red circles) across included studies. Each point represents single reported value from the literature. Time since implantation is shown in months and concentration is reported as micrograms per liter (µg/L)

Scatter of Reported Data for Titanium

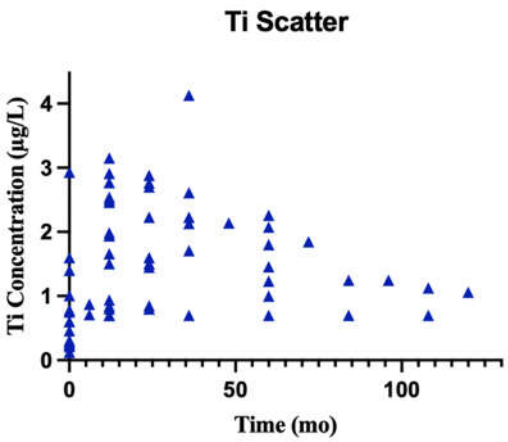

Figure S2. Reported concentrations titanium measured in serum and across studies. Each point represents single reported value from the literature. Time since implantation is shown in months and concentration is reported as micrograms per liter (µg/L)

Supplemental Analysis: Random Data Simulation of Serum Ion Trajectories

These random data simulations were attempted under the assumption of linear behavior to illustrate how pooled data might appear if temporal changes were considered in a purely linear framework. However, this approach does not represent actual kinetics, as clinical data consistently demonstrate a rapid early postoperative rise better captured by previous models.

A total of 500 data points were randomly generated across a postoperative timeline in months, using normal distributions parameterized by pooled means and standard deviations reported in the literature. Each simulated value therefore reflects a possible patient-level metal ion concentration around those reported summary statistics. Because this approach assumes normality and independence across timepoints, it does not reconstruct individual trajectories but instead illustrates hypothetical population-level dispersion. In some cases, this assumption also produced postoperative concentrations at zero, which should be interpreted as artifacts of the simulation rather than physiological values.

Random Generated Data for Cobalt

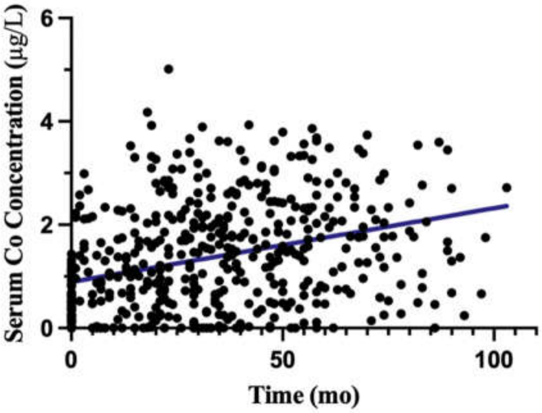

Figure S3. Simulated serum cobalt (Co) concentrations over time following THA. A total of 500 values were generated using normal distributions by pooled means and standard deviations from the literature. Each point represents a simulated patient-level concentration across a postoperative timeline. Linear regression of the simulated dataset revealed a weak but statistically significant positive trend (slope = 0.0140 μg/L per month, 95% CI: 0.0104–0.018, $p < 0.0001$, $R^2 = 0.11$). Pearson correlation confirmed this association ($r = 0.323$, 95% CI: 0.243–0.400, $p < 0.0001$). While the regression line indicates a slow upward trajectory in predicted concentrations, the broad scatter illustrates high inter-patient variability, with values ranging from near baseline to >4 μg/L at similar timepoints.

Random Generated Data for Chromium

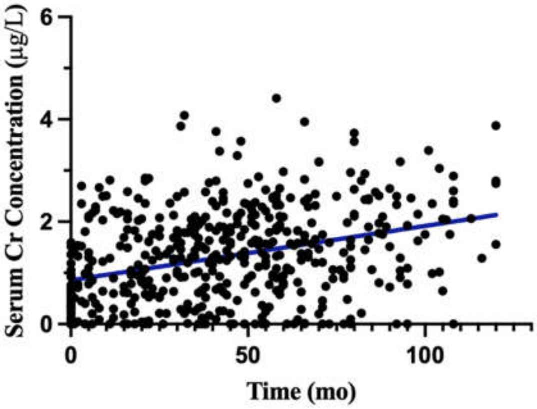

Figure S4. Simulated serum chromium (Cr) concentrations over time following THA. A total of 500 data points were generated using normal distributions by pooled means and standard deviations from the literature. Linear regression of the simulated dataset yielded a slope of 0.011 µg/L per month (95% CI: 0.008–0.013), and $R^2 = 0.12$. Pearson's correlation indicated a weak positive association between time and chromium concentration ($r = 0.34$, 95% CI: 0.27–0.42, $p < 0.0001$). While the regression line suggests a gradual rise in simulated concentrations over time, the wide scatter illustrates the high degree of inter-patient variability that characterizes chromium kinetics.

Random Generated Data for Titanium

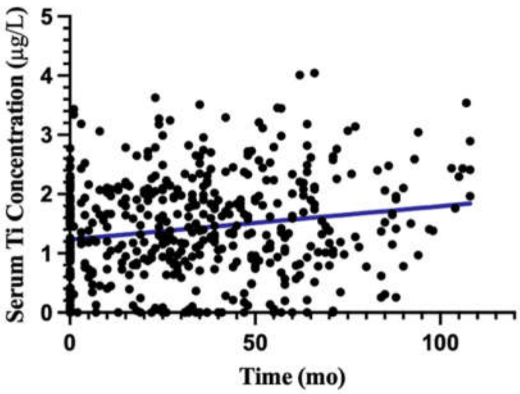

Figure S4. Simulated serum titanium (Ti) concentrations over time following THA. A total of 500 data points were generated using normal distributions by published study means and standard deviations to approximate patient-level variability. Each point represents a possible serum Ti concentration across a postoperative window. Linear regression of the simulated dataset demonstrated a weak but statistically significant positive slope (0.0056 µg/L per month, 95% CI: 0.0029–0.0083, $p < 0.0001$, $R^2 = 0.033$). Pearson correlation confirmed this weak association ($r = 0.18$, 95% CI: 0.095–0.265, $p < 0.0001$). While the regression line suggests only minimal accumulation of titanium over time, the broad scatter illustrates pronounced inter-patient variability.

Random Generated Data for Molybdenum

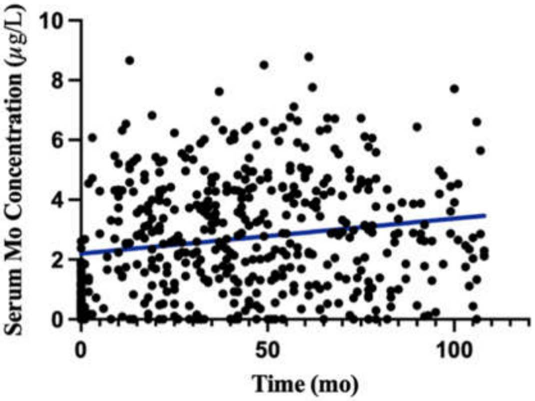

Figure S4. Simulated serum molybdenum (Mo) concentrations over time following THA**.** A total of 500 values were generated using normal distributions by pooled means and standard deviations from the literature. Each point represents a simulated patient-level concentration across a postoperative timeline. Linear regression of the simulated dataset revealed a weak but statistically significant positive slope (0.0117 µg/L per month, 95% CI: 0.0060–0.0175, $p < 0.0001$, $R^2 = 0.031$). Pearson's correlation confirmed this association ($r = 0.18$, 95% CI: 0.09–0.26, $p < 0.0001$). While the regression line indicates a gradual increase in simulated Mo levels, the wide scatter illustrates the high degree of inter-patient variability that characterizes Mo kinetics.

Random Generated Data for Nickel

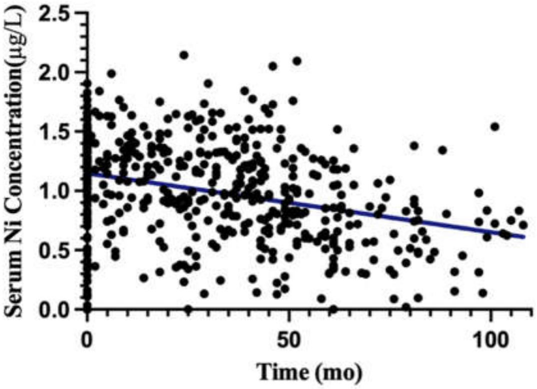

Figure S5. Simulated serum nickel (Ni) concentrations over time following THA**.** A total of 500 values were generated using normal distributions by pooled means and standard deviations from the literature. Linear regression of the simulated dataset revealed a significant negative slope (−0.0050 µg/L per month, 95% CI: −0.0063 to −0.0036, $p < 0.0001$, $R^2 = 0.095$), indicating a modest decline in concentrations with longer follow-up. Pearson's correlation confirmed this association ($r = -0.31$, 95% CI: −0.39 to −0.23, $p < 0.0001$). The downward trajectory was driven in part by limited long-term data, with one study reporting low concentrations beyond 100 months, and may also reflect improvements in implant metallurgy that reduce nickel release. The wide scatter highlights high inter-patient variability despite the overall trend.

Note: random nickel data generation may be skewed due to limited long-term Ni data, may not reflect trends in general population
